# Synbiotic yoghurt with walnut and cereal brittle added as a next‐generation bioactive compound: Development and characteristics

**DOI:** 10.1002/fsn3.1135

**Published:** 2019-07-25

**Authors:** Ivan Fiodorovich Gorlov, Valeria Viktorovna Shishova, Marina Ivanovna Slozhenkina, Olga Petrovna Serova, Natalia Ivanovna Mosolova, Elena Yurievna Zlobina, Tatiana Nikolaevna Barmina

**Affiliations:** ^1^ Volga region research institute of manufacture and processing of meat‐and‐milk production Volgograd Russia; ^2^ Volgograd State Technical University Volgograd Russia; ^3^ Volgograd State University Volgograd Russia

**Keywords:** bioavailability, meal plan models, micronutrient deficiency, nutrient intake, scaling up nutrition, supplementation

## Abstract

The article presents a technology developed for the production of synbiotic Yoghurt with new bioactive filler based on natural components. The Yoghurt has prebiotic and sorption properties. A higher consumer appeal of the product developed has been substantiated; its characteristics compared with the Yoghurt of traditional production technology have been presented. The brittle, containing peeled walnuts, as well as barley, wheat, rye, oatmeal and buckwheat flakes, sugar, and water, was used as a filler. Optimum time and temperature regimes of boiling caramel mixtures and brewing raw walnut–cereal mass in the brittle have been selected. The formulation developed enables increasing the nutritional and biological values of the finished product. The research studies of the finished product involved an analysis of organoleptic, physicochemical, and microbiological points. When performing the tasks, the approved regulatory and technical documentation (GOST) was applied. Each measurement was carried out in triplicate. The physicochemical characteristics of the samples developed were compared with the requirements for the quality of fermented milk products. The nutritional and biological values were calculated. The increase in consumer properties, and nutritional and biological values of the finished dairy product was scientifically substantiated and experimentally confirmed.

**Highlights**

- A higher consumer appeal of the product developed has been substantiated; its characteristics compared with the Yoghurt of traditional production technology have been presented.

## 1 INTRODUCTION

For millennia, microbes have been used in the production of food and beverages; the technology involves the fermentation process. In recent years, the attention of scientists has been attracted by identifying fundamental mechanisms and influence patterns of food and beverages on the human body, and as a consequence, the options of their use in the food industry for preventive and therapeutic purposes. As a result, the world community has got a new term “probiotics.” Lactic acid bacteria (LAB) are normal microflora in the intestines of mammals (Markowiak & Slizewska, 2018; Young, 1998). They play an important role in the process of vital activity and act as an immunomodulator. The LAB are widely used for prevention and treatment of gastrointestinal diseases, as well as for improvement of digestion and more complete absorption of nutrients. Probiotic microorganisms include lactic acid bacteria such as *Lactobacillus acidophilus, L. bulgaricus*, *L. casei*, *L. plantarum*, *and L. rhamnosus*. These applied live bacteria promote the development of the immune response and distribution of the vaccine component in the body. Complexes of pathogenic and nonpathogenic food microbes are currently evaluated as candidates for oral vaccines. Intestinal microflora plays a crucial role in the metabolism of various nutrient substrates in the vital activity. There are numerous studies of pre‐, pro‐, and synbiotics, confirming clinical efficiency in maintaining the balance of the gastrointestinal microbiota to improve health (Nakagawa, Yabuuchi, Yasokawa, & Nagashima, 2005; Quigley, 2019; Singh, Amdekar, Yadav, Mishra, & Jain, 2011; Sukhikh, Krumlikov, Evsukova, & Asyakina, 2017; Vyas & Ranganathan, 2012). Prebiotics are known to be food components that are not digested and absorbed in the upper gastrointestinal tract, since gastric enzymes cannot act on them; but they are fermented by the microflora of the human large intestine and stimulate its growth and vital activity. They reach the colon intact and are selectively fermented to have a beneficial effect. Probiotics are live cultures of microorganisms and substances of microbial and other origin. Synbiotics are combined bacterial preparations containing prebiotics and probiotics and belong to the innovation group of biologics (Mohanty, Mishra, Mohapatra, & Sahu, 2018; Roberfroid, 2000; de Vrese & Schrezenmeir, 2008). A bioactive compound is a simple substance that has a biological activity associated with its ability to modulate one or more metabolic processes, which leads to improved health (Angiolillo, Nobile, & Conte, 2015; Watson & Preedy, 2015).

One of the most important tasks in addressing the food safety and nutrition issues and achieving the goals of sustainable development is the elimination of all forms of malnutrition (FAO et al., 2017). In 2016, the decade of the UN action on nutrition began (2016–2025).

In Russia, in the framework of the Order “On basic principles of state policy in the field of healthy nutrition of the population of the Russian Federation for the period of up to 2020,” there were some improvements noted in the area of nutrition of the population due to changes in the structure of food consumption, that is, increasing the share of specialized meat and dairy products (The order of the Government of the Russian Federation dated 25.10.10). So, obvious is the relevance of expanding the range of functional fermented milk products that improve digestion, quickly eliminate hunger, and are a source of micro‐ and macronutrients (McAuliffe, Kilcawley, & Stefanovic, 2019; Slozhenkina, Druker, Kryuchkova, & Zlobina, 2018; Slozhenkina, Serova, Vodolazkova, & Zlobina, 2017). Among the variety of dairy products, Yoghurts can be considered the most common and attractive for the consumer; their advantages, in particular, include light texture, pleasant organoleptic characteristics, and various fillers and functional ingredients that are able to be added to the production technology and increase the biological and nutritional value of the product (Mohammadi‐Sartang et al., 2018; Sivieri et al., 2017). Nontraditional components such as inulin (Mazloomi, Shekarforoush, Ebrahimnejad, & Sajedianfard, 2011), fructooligosaccharides (Madhu, Amrutha, & Prapulla, 2012), oat/barley beta‐glucan (Ladjevardi, Yarmand, Emam‐Djomeh, & Niasari‐Naslaji, 2016; Vasiljevic, Kealy, & Mishra, 2007), different kinds of rice (Kumari, Ranadheera, Prasanna, Senevirathne, & Vidanarachchi, 2015; Wattananapakasem, Valenberg, Fogliano, Costabile, & Suwannaporn, 2018), pineapple peel powder (Sah, Vasiljevic, McKechnie, & Donkor, 2016), Lesser Yam Tubers (*Dioscoreaesculenta *L.) (Winarti & Saputro, 2015), probiotics and prebiotics (Hill, Ross, Arendt, & Stanton, 2017), red ginger extract (Larasati, Panunggal, Afifah, Anjani, & Rustanti, 2017), Binahong Leaf Extract (Lestari, Nissa, Afifah, Anjani, & Rustanti, 2017), and Purple Sweet Potato (Tari, Handayani, & Hartati, 2018) are known to be used as fillers for the production of synbiotic yoghurt.

**Figure 1:**
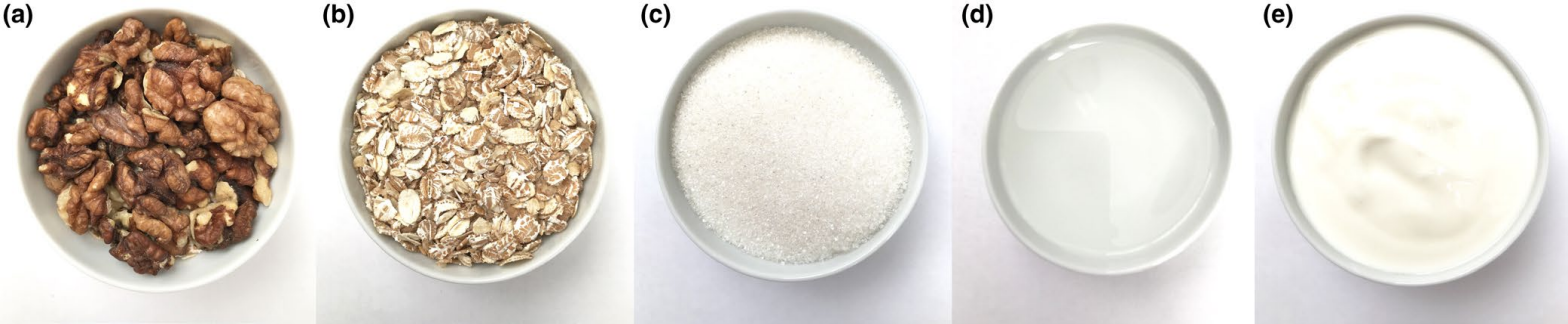
Raw materials for enriched product: (a) walnuts; (b) cereal flakes; (c) sugar; (d) water; and (e) milk

The authors proposed a recipe and developed a technology for the production of a new synbiotic Yoghurt using a nontraditional filler, that is, brittle of walnut and five cereal flakes (barley, wheat, rye, oats, and buckwheat) as a prebiotic component and plant sorbent (Figure 1).

## 2 MATERIALS AND METHODS

### 2.1 The samples of raw materials and the production technology

**Figure 2:**
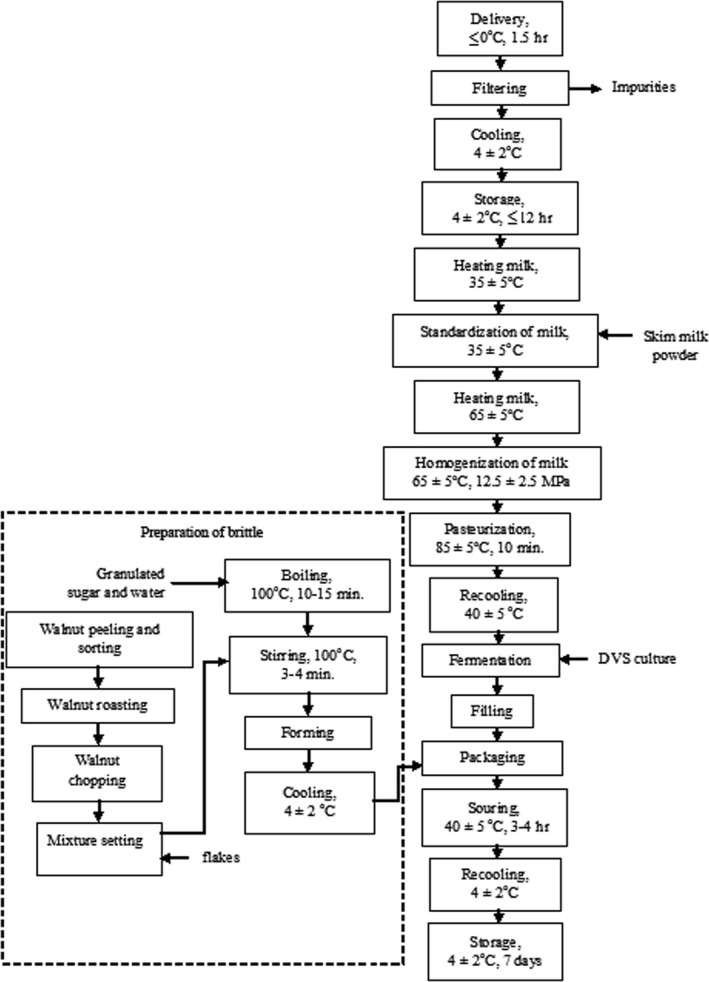
Technological scheme for the product enriched (thermostatic method)

The set yoghurt was produced in accordance with the traditional technological scheme (Tamime & Robinson, 2007) that includes the following steps: delivery of raw materials; filtering, cooling, and storage; heating, standardization, and homogenization; pasteurization and recooling; fermentation; filling, packaging, and labeling; souring and recooling; and storage (Figure 2 and Table 1).

**Table 1:** Recipe of synbiotic Yoghurt with filler

Raw material	Amount, kg per 1,000 kg
Fermented milk base	
Standardized milk	923.0
Skim milk powder	76.9
Culture	0.1
Total	1,000.0
Filler	
Walnuts	277.0
Five cereal flakes	277.0
Granulated sugar	277.0
Water	169.0
Total	1,000.0
Fermented milk base/filler, %	70/30

At the fermentation stage, dry bacterial starter culture was used to produce dairy products based on *Lactobacillus bulgaricus, Lactobacillus casei,* and *Streptococcus thermophilus* (LbS 22.11 series, “AiBi,” OOO “Green Lines,” Russia). AiBi direct application starters are highly concentrated lyophilized cultures containing a mixture of precisely defined strains that provide effective and reliable development of the acidity, good texture of the product, precisely defined production process, and exactly determined time of ripening. They are phage‐alternative and phage‐resistant for a long time.

Brittle was chosen as bioactive filler with prebiotic and adsorbing properties; there was a developed formulation that included peeled walnuts, flakes of wheat, rye, oat, barley and buckwheat, sugar, and water. All brittle samples were produced according to one recipe, except for Test I sample added with citric acid (5 g per 100 g of the mixture). Three brittle samples were developed, that is, Control with low melting point; Test I with citric acid, and Test II with high melting point.

The basic scheme of the brittle production included the operations of raw material preparation, syrup boiling, walnut–cereal mass brewing, forming, cooling, and storage (Figure 2 and Table 1).

Optimum time and temperature conditions for boiling caramel mixtures and brewing raw walnut–cereal masses in caramel were determined using a single‐block logic programmable controller OVEN PLK 150–220.I‐M (OOO Production Association OVEN, Russia) and the CODESYS v. 2 programming system (CODESYS Group, 3S‐Smart Software Solutions GmbH, Germany).

**Figure 3:**
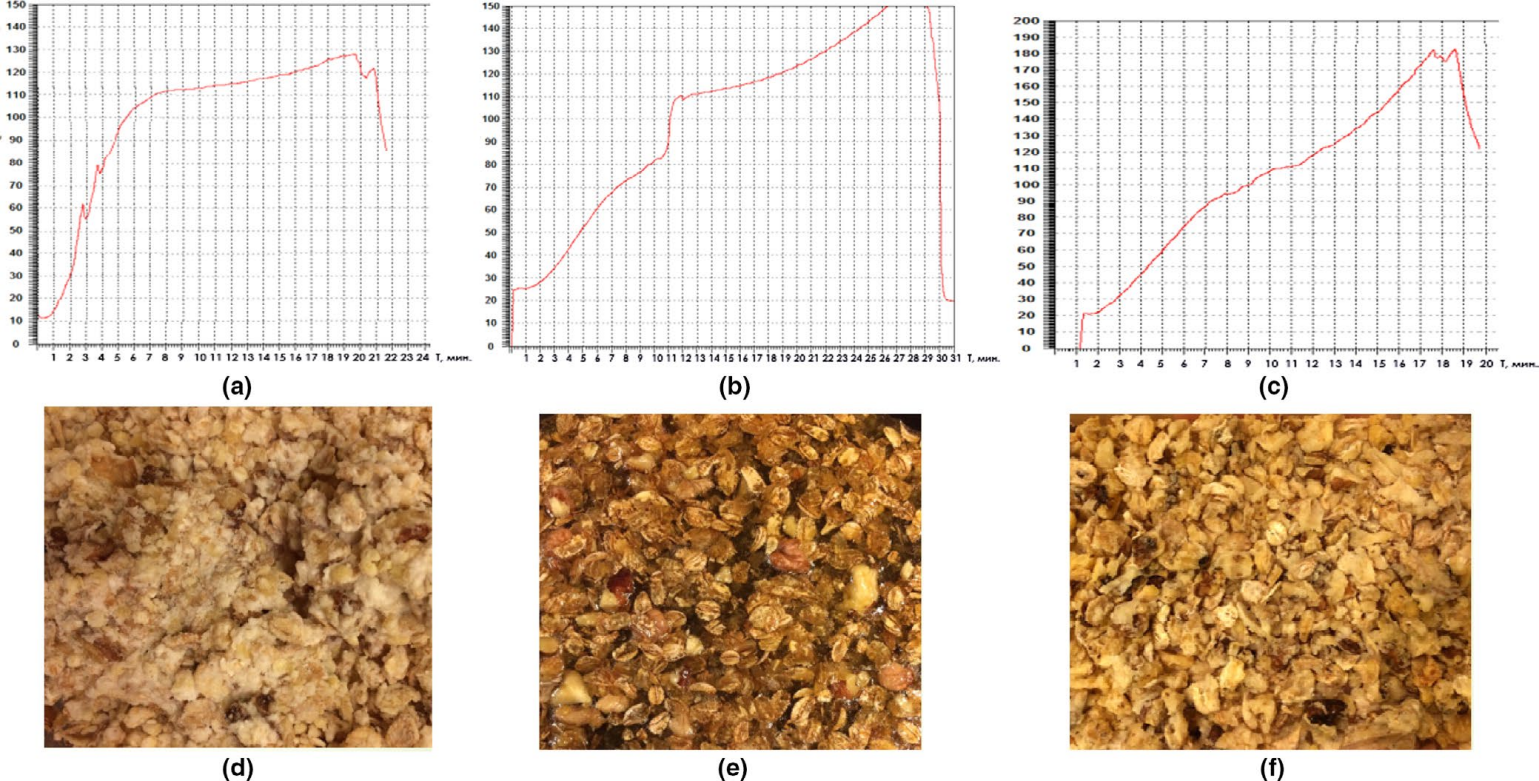
Temperature and time curves of the boiling process of caramel mixture and the structure of the brittle samples: (a, d) Control; (b, e) Test I; (c, f) Test II

The experimental study to establish the optimum temperature and time of melting the caramel mixture was carried out as follows. The sugar syrup was stirred and boiled to a moisture content of 4%–6% at a temperature of 100–180°C (Table 2). Then, premixed walnuts and cereals were poured into the syrup and mixed. The walnut–cereal mass was molded and cooled to 20°C. In the metal container, there was a temperature sensor preinstalled and completely immersed in the area of the caramel mixture during the experiment. The sensor was connected to a monoblock controller with discrete and analogue inputs/outputs on board for the OVEN PLK 150 small systems automation; the temperature and time regimes were recorded continuously throughout all the preparation stages. The controller was programmed by the professional programming system CODESYS v.2. This program helped output the information to a computer screen; all processes were presented in a temperature–time curve (Figure 3a‐c).

**Table 2:** Temperature and time regimes of melting and brewing caramel, walnut, and cereal mass

Process stage	Temperature, ^o^C			Time, min			Description
	Control	Test I	Test II	Control	Test I	Test II	
Boiling the caramel mass	70	73	75	4–5	8	5–6	Start of melting
	110	90	90	7–8	10–11	7	Start of boiling
	119	115	125	13	15	12	Boiling the mixture
	126	142	158	17	25	15	Homogeneous mixture
	127	161	176	21–22	27	17	Darkening of the mass
Brewing the nut–cereal mass	127	170	180	22	28	18	Brewing
	80	110	110	23	29	19	Homogeneous mixture

### 2.2 Analysis of the finished product characteristics

The research studies of the finished product involved an analysis of organoleptic, physicochemical, and microbiological points. When performing the tasks, the approved regulatory and technical documentation (GOST) was applied. Each measurement was carried out in triplicate. The physicochemical characteristics of the samples developed were compared with the requirements for the quality of fermented milk products.

Technical regulations of the Customs Union “On safety of milk and dairy products” (TR TS 033/2013) and GOST 31981–2013 “Yoghurts. General specifications.” Sensory analysis of the developed enriched products was carried out in accordance with GOST R ISO 22935–2–2011 “Milk and milk products” and GOST R ISO 22935–3–2011 “Milk and milk products. Sensory analysis. Part 3. Guidance on a method for evaluation of compliance with product specifications for sensory properties by scoring” (a five‐point scale applied). Microbiological characteristics of the developed milk products were determined in accordance with the requirements of GOST 32901–2014 “Milk and milk products. Methods of microbiological analysis.” The amino acid composition was analyzed by a HPLC analyzer for physiological fluids with postcolumn ninhydrin derivatization in accordance with the manufacturer's instruction manual (ARACUS, PMA GmbH, Germany). Photographs of the brittle samples structures were taken using the Mikmed‐5 light microscope (AO LOMO, Russia), 10× magnification.

### 2.3 The calculation of the nutritional and biological values

The nutritional value (NV) was evaluated in accordance with the following formula:

$$\frac{\text{NV}\;(\text{kcal)}}{100\;\text{g}}=4\cdot P+9\cdot F+4\cdot C$$

where *P* is the percentage of protein, %; *F* is the percentage of fat, %; *C* is the percentage of carbohydrates, %.

The amino acid score (AAS) was calculated as follows:

$$\text{AAS}\;\left(\%\right)=\frac{\text{Evaluated}\;\text{protein}\;\text{amino}\;\text{acid}\;\text{content}\;(\text{g}/100\;\text{g}\;\text{protein})}{\text{FAO}\;\text{scoring}\;\text{model}\;\text{amino}\;\text{acid}\;\text{content}\;(\text{g}/100\;\text{g}\;\text{protein})}\times 100$$

The coefficient of amino acid score difference (CAAS) was calculated by the following formula:

$$\text{CAAS}\;\left(\%\right)=\frac{\sum (C_{i}-C_{\min})}{n}$$

where *C_i_* is the excess of amino acid score, %; *C*_min_ is the minimum score of essential amino acids of evaluated protein relative to the physiological norm (FAO scoring model amino acid content, %)

$$\sum (C_{i}-C_{\min})=\Delta \text{AAS}$$

*n* is the number of essential amino acids.

The biological value (BV) was calculated by the following formula:

BV(%)=100-CAAS

To assess the balance of essential amino acids with respect to the reference protein, the utility *K_i_* coefficient was calculated.

$$K_{i}=\frac{{\text{AS}}_{\min}}{{\text{AS}}_{i}}$$

where AS*_i_* is the amino acid score of the i‐indispensable amino acid in the product; AS_min_ is the amino acid score of the first limiting amino acid in this product.

The utility coefficient of the *i*‐indispensable amino acid (*A*_i_) was used to calculate the utility coefficient of the amino acid composition (*U*), which was a numerical characteristic that reflected the balance of essential amino acids with respect to the standard (*A_i_*st):

$$U=\frac{\sum_{i=1}^{n}\left(A_{i}K_{i}\right)}{\sum_{i=1}^{n}A_{i}\text{st}}.$$

### 2.4 Statistical analysis

The experimental data were analyzed by the Statistica 10.0 package using conventional techniques. The threshold of 0.05 was considered a minimum threshold of the significance of differences (Johnson & Bhattacharyya, 2010). The MS Office 2010 was used for the graphical interpretation of the digital data.

## 3 RESULTS AND DISCUSSION

### 3.1 Comparative quality evaluation of the brittle samples

**Figure 4:**
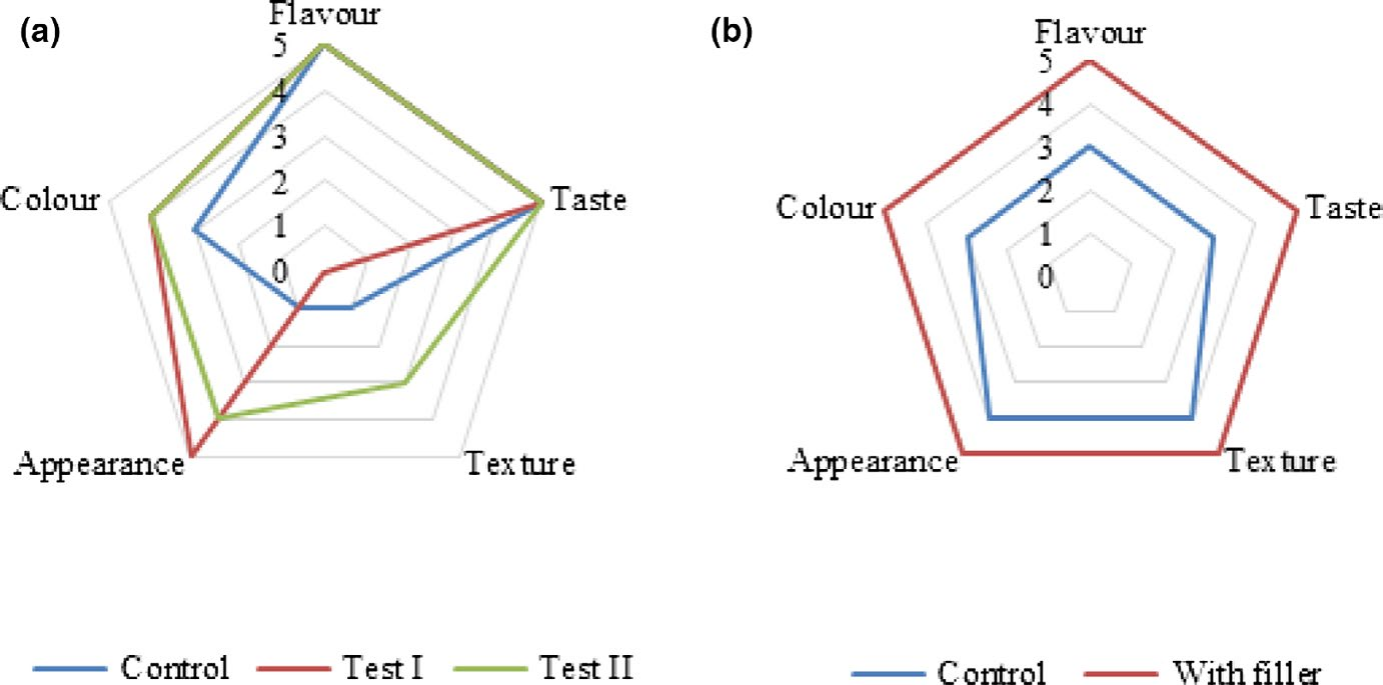
Profilograms of the samples: (a) brittle; (b) finished product

The Control brittle sample was made at low melting point of the caramel mass (up to 127°C); the moisture content in the finished product exceeded the similar value in the Test ones by 4.99% (*p *< .01) and 2.97% (*p* < .05), respectively. The Control sample was inferior to the Test ones (Table 3). So, the consistency of the Control sample was too short‐brittle due to the insufficient caramelization degree (Figure 3d). The higher moisture content in the Control sample led to a lower content of macronutrients. So, the protein content was lower by 0.13 (*p* < .05) and 0.08% (*p* < .05); fat content by 0.81 (*p* < .05) and 0.41% (ns); and carbohydrate content by 1.49 (*p* < .05) and 0.87% (ns), respectively. In this regard, the Control was characterized by a minimum nutritional value in comparison with its analogues (by 3.62 and 1.96%, respectively). Taking into account the defects of the Control sample caused by insufficient heating, the caramelization temperature of the Test I sample was brought to 170°C. However, despite the fact that this sample was characterized by the highest content of macronutrients, by organoleptic characteristics it was inferior to the Control and Test II samples in too hard and dry consistency and unpleasant dark brown color (Figure 3e). Moreover, citric acid in its composition caused sour taste. The Test II sample was prepared at higher melting point of the caramel mass (up to 180°C), without citric acid added, which made it possible to obtain a sample with the most attractive organoleptic and physicochemical characteristics (Figure 3f). Thus, the most attractive brittle characteristics were found to be achieved without citric acid, at the melting temperature of the caramel mixture of 100–180°C and in‐process time of 19 min (Table 2 and Figures 3 and 4).

**Table 3:** Characteristics of the brittle samples (mean ± *SEM*)

Parameter	Control	Test I	Test II
Dry matter, %	91.17 ± 0.74	96.16 ± 0.62**	94.14 ± 0.68*
Protein, %	6.95 ± 0.02	7.08 ± 0.03*	7.03 ± 0.02*
Fat, %	17.21 ± 0.13	18.02 ± 0.17*	17.62 ± 0.09^ns^
Carbohydrates, %	48.94 ± 0.32	50.43 ± 0.26*	49.81 ± 0.23^ns^
Nutritional value, kcal/100 g	378.5	392.2	385.9

Abbreviation: ns, not significant at *p* > 0.05 compared with data on the Control sample. ***p < .001.

* *p* < .05.

** *p* < .01.

### 3.2 Comparative quality evaluation the of finished product samples

**Figure 5:**
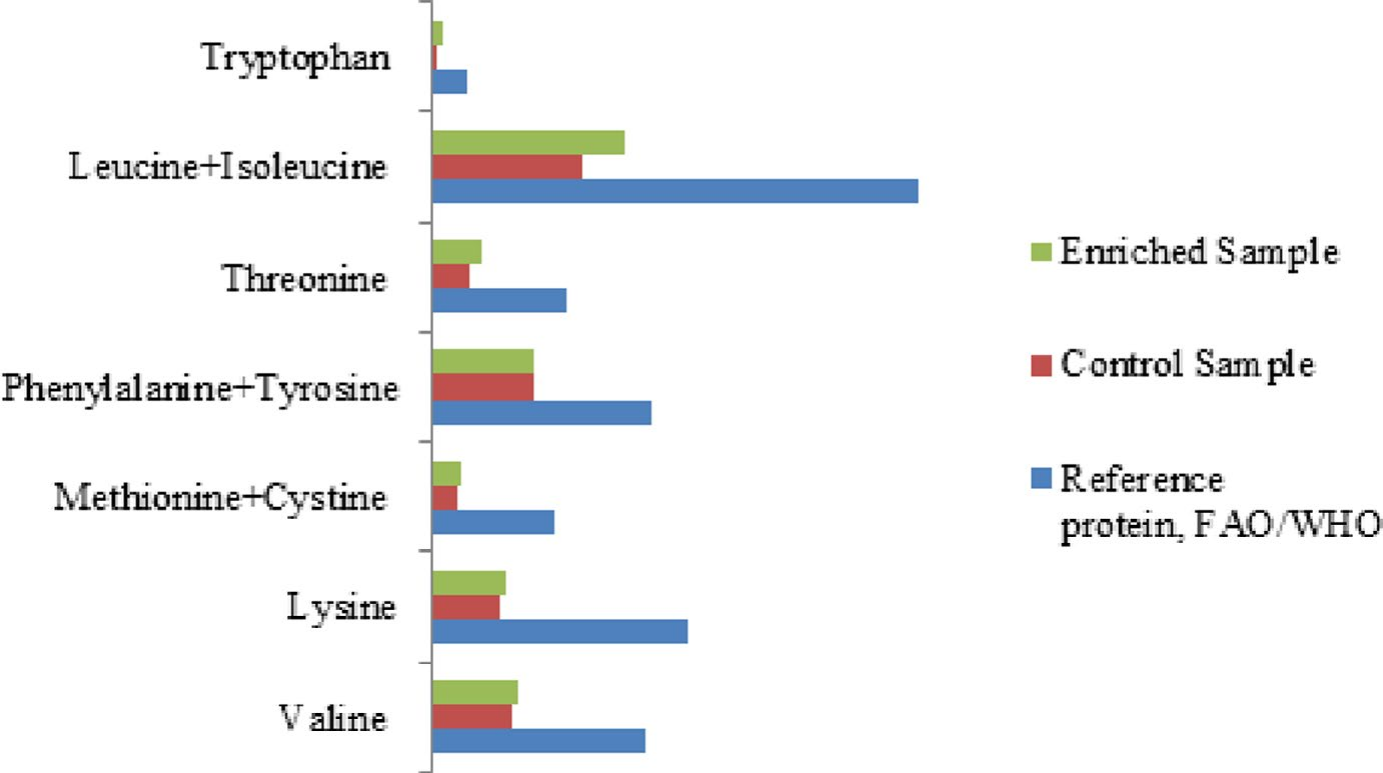
Amino acid composition of the samples in comparison with the reference protein (FAO/WHO), mg per 1 g of protein

To evaluate the quality of the finished product, a brittle Yoghurt sample (brittle was precrushed and mixed with Yoghurt) was compared with the Yoghurt sample produced according to conventional technology (Tamime & Robinson, 2007). Comparative analysis of the Control sample and the enriched one is given in Table 4. So, the introduction of a filler in Yoghurt allowed increasing the content of dry substances in it by 24.21% (*p* < .001); protein by 1.12% (*p* < .001); fat by 4.18% (*p* < .001); and carbohydrates by 13.53% (*p* < .001). The nutritional value of the product developed was 161.5 kcal per 100 g. According to the microbiological points, the samples developed met the requirements of the Technical Regulations of the Customs Union “On the safety of milk and dairy products” (TR CU 033/2013). The organoleptic analysis made it possible to design a profilogram (Figure 4). This analysis also revealed the superiority of the enriched product over the Control sample. So, this study found a positive effect of the filler developed on the amino acid composition of Yoghurt and its biological value (Table 5 and Figure 5).

**Table 4:** Comparative characteristics of the Yoghurt samples

Parameter	Control (conventional technology)	Test (with filler)
Dry matter, %	13.44 ± 0.06	37.65 ± 2.44*
Protein, %	3.29 ± 0.05	4.41 ± 0.08*
Fat, %	3.69 ± 0.03	7.87 ± 0.04*
Carbohydrates, %	4.72 ± 0.04	18.25 ± 0.11*
Nutritional value, kcal/100 g	65.3	161.5

Abbreviation: ns, not significant at *p* > .05 compared with data on the Control sample. **p < .01; ***p < .05.

* *p* < .001.

**Table 5:** Indices of the biological value of the Yoghurt samples

Amino acids, mg per 1 g of protein	Control Sample								Enriched Sample								Reference protein, FAO/WHO
	*A_i_*	AAS,%	ΔAAS	CAAS,%	BV	*K_i_*	A_i_* K_i_	*U*	*A_i_*	AAS,%	ΔAAS	CAAS,%	BV	*K_i_*	A_i_* K_i_	*U*	
Valine	14.8	37.03	21.87			0.41	6.06		15.90	39.75	15.40			0.61	9.74		40.0
Lysine	12.9	26.88	11.72			0.56	7.27		13.60	28.33	3.99			0.86	11.69		48.0
Methionine + Cystine	4.9	21.35	6.20			0.71	3.48		5.60	24.35	0.00			1.00	5.60		23.0
Phenylalanine + Tyrosine	18.8	45.88	30.73	14.31	85.69	0.33	6.21	0.475	19.10	46.59	22.24	10.63	89.37	0.52	9.98	0.659	41.0
Threonine	7.3	29.36	14.21			0.52	3.79		9.40	37.60	13.25			0.65	6.09		25.0
Leucine + Isoleucine	27.8	30.56	15.41			0.50	13.79		35.90	39.45	15.10			0.62	22.16		91.0
Tryptophan	1.0	15.15	0.00			1.00	1.00		1.90	28.79	4.44			0.85	1.61		6.6

Abbreviations: AAS, an amino acid score; BV, a biological value; CAAS, a coefficient of amino acid score difference; *K_i_*, an utility coefficient; *U*, an utility coefficient of the amino acid composition.

As the evaluation showed (Table 6), the formation of lactic acid microorganisms was different in yoghurt samples enriched with bioactive filler (Test) and not enriched (Control). The brittle in Yoghurt influenced the growth and reproduction of lactic microflora.

**Table 6:** Microbiological indicators of Yoghurt samples

Indicator	Technical regulations of the customs union 033/2013	Storage period, days									
		1		3		5		7		9	
		Test	Control	Test	Control	Test	Control	Test	Control	Test	Control
CFU/cm^3^ (g)	Lactic acid microorganisms, not less 1 × 10^7^	1.3 × 10^9^	1.1 × 10^9^	3.9 × 10^9^	1.3 × 10^9^	2.3 × 10^9^	1.9 × 10^9^	1.4 × 10^9^	4.3 × 10^8^	1.5 × 10^8^	1.2 × 10^7^
Weight of the product that does not allow, g:											
Coliforms	0.01	Not determined									
Yeast, CFU/cm^3^ (g), not more than	50	Not determined									
Mold, CFU/cm^3^ (g), not more than	50	Not determined									

In the industrial Yoghurt production according to the technology developed, the finished product was proposed to be packed in two‐section plastic cups. Yoghurt and filler should be mixed before eating by the consumer.

## 4 CONCLUSIONS

The technology was developed for the production of a new synbiotic Yoghurt with a plant filler in form of brittle with prebiotic and sorption properties. The brittle formulation was developed; in the course of laboratory studies using specialized hardware support, the temperature and time regimes of its production were optimized. The increase in consumer properties, and nutritional and biological values of the finished dairy product was scientifically substantiated and experimentally confirmed.

## CONFLICT OF INTEREST

The authors declare that they do not have any conflict of interest.

## ETHICAL APPROVAL

This study does not involve any human or animal testing.

## INFORMED CONSENT

Written informed consent was obtained from all study participants.

## References

[fsn31135-bib-0001] Angiolillo, L. , Del Nobile, M. A. , & Conte, A. (2015). The extraction of bioactive compounds from food residues using microwaves. Current Opinion in Food Science, 5, 93–98. 10.1016/j.cofs.2015.10.001

[fsn31135-bib-0002] de Vrese, M. , & Schrezenmeir, J. (2008). Probiotics, prebiotics, and synbiotics. Food Biotechnology, 111, 1–66. 10.1007/10_2008_097 18461293

[fsn31135-bib-0003] FAO , IFAD , UNICEF , WFP & WHO (2017). The State of Food Security and Nutrition in the World. Building resilience for peace and food security. Rome: FAO.

[fsn31135-bib-0004] Hill, D. , Ross, R. P. , Arendt, E. , & Stanton, C. (2017). Microbiology of yogurt and bio‐yogurts containing probiotics and prebiotics. Yogurt in Health and Disease Prevention, 69–85.

[fsn31135-bib-0005] Johnson, R. A. , & Bhattacharyya, G. K. (2010). Statistics principles and methods (6th ed.). USA: John Wiley & Sons.

[fsn31135-bib-0006] Kumari, A. , Ranadheera, C. S. , Prasanna, P. H. P. , Senevirathne, N. D. , & Vidanarachchi, J. K. (2015). Development of a rice incorporated synbiotic yogurt with low retrogradation properties. International Food Research Journal, 22, 2032–2040.

[fsn31135-bib-0007] Ladjevardi, Z. S. , Yarmand, M. S. , Emam‐Djomeh, Z. , & Niasari‐Naslaji, A. (2016). Physicochemical Properties and viability of probiotic bacteria of functional synbiotic camel yogurt affected by oat beta‐glucan during storage. Journal of Agricultural Science and Technology, 18, 1233–1246.

[fsn31135-bib-0008] Larasati, B. A. , Panunggal, B. , Afifah, D. N. , Anjani, G. , & Rustanti, N. (2017). Total lactic acid bacteria, antioxidant activity, and acceptance of synbiotic yoghurt with red ginger extract (Zingiberofficinale var. rubrum). 3rd International Conference on Tropical and Coastal Region Eco Development (ICTCRED). Yogyakarta, Indonesia.

[fsn31135-bib-0009] Lestari, R. P. , Nissa, C. , Afifah, D. N. , Anjani, G. , & Rustanti, N. (2017). Total Lactic Acid Bacteria (LAB), Antioxidant Activity, and Acceptance of Synbiotic Yoghurt with Binahong Leaf Extract (Anredera cordifolia (Ten.) Steenis). 3rd International Conference on Tropical and Coastal Region Eco Development (ICTCRED). Yogyakarta, Indonesia

[fsn31135-bib-0010] Madhu, A. N. , Amrutha, N. , & Prapulla, S. G. (2012). Characterization and antioxidant property of probiotic and synbiotic yogurts. Probiotics and Antimicrobial Proteins, 4, 90–97. 10.1007/s12602-012-9099-6 26781850

[fsn31135-bib-0011] Markowiak, P. , & Slizewska, K. (2018). The role of probiotics, prebiotics and synbiotics in animal nutrition. Gut Pathogens, 10 10.1186/s13099-018-0250-0 29930711 PMC5989473

[fsn31135-bib-0012] Mazloomi, S. M. , Shekarforoush, S. S. , Ebrahimnejad, H. , & Sajedianfard, J. (2011). Effect of adding inulin on microbial and physico‐chemical properties of low fat probiotic yogurt. Iranian Journal of Veterinary Research, 12, 93–98.

[fsn31135-bib-0013] McAuliffe, O. , Kilcawley, K. , & Stefanovic, E. (2019). Symposium review: Genomic investigations of flavor formation by dairy microbiota. Journal of Dairy Science, 102, 909–922. 10.3168/jds.2018-15385 30343908

[fsn31135-bib-0014] Mohammadi‐Sartang, M. , Bellissimo, N. , de Zepetnek, J. O. T. , Brett, N. R. , Mazloomi, S. M. , Fararouie, M. , … Mazloom, Z. (2018). The effect of daily fortified yogurt consumption on weight loss in adults with metabolic syndrome: A 10‐week randomized controlled trial. Nutrition Metabolism and Cardiovascular Diseases, 28, 565–574. 10.1016/j.numecd.2018.03.001 29724529

[fsn31135-bib-0015] Mohanty, D. , Mishra, S. , Mohapatra, S. , & Sahu, P. S. (2018). Prebiotics and synbiotics: Recent concepts in nutrition. Food Bioscience, 26, 152–160. 10.1016/j.fbio.2018.10.008

[fsn31135-bib-0016] Nakagawa, R. , Yabuuchi, H. , Yasokawa, D. , & Nagashima, K. (2005). Fermentation of soybean milk with Lactobacillus plantarum Hokkaido and its health function. Journal of the Japanese Society for Food Science and Technology‐Nippon Shokuhin Kagaku Kogaku Kaishi, 52, 140–143. 10.3136/nskkk.52.140

[fsn31135-bib-0017] Quigley, E. M. M. (2019). Prebiotics and Probiotics in Digestive Health. Clinical Gastroenterology and Hepatology, 17, 333–344. 10.1016/j.cgh.2018.09.028 30267869

[fsn31135-bib-0018] Roberfroid, M. B. (2000). Prebiotics and probiotics: Are they functional foods? American Journal of Clinical Nutrition, 71, 1682S–1687S. 10.1093/ajcn/71.6.1682S 10837317

[fsn31135-bib-0019] Sah, B. N. P. , Vasiljevic, T. , McKechnie, S. , & Donkor, O. N. (2016). Physicochemical, textural and rheological properties of probiotic yogurt fortified with fibre‐rich pineapple peel powder during refrigerated storage. LWT‐Food Science and Technology, 65, 978–986. 10.1016/j.lwt.2015.09.027

[fsn31135-bib-0020] Singh, V. , Amdekar, S. , Yadav, H. , Mishra, N. N. , & Jain, S. (2011). Future Application of Probiotics: A Boon from Dairy Biology (pp. 87–100). Microbes and Microbial Technology: Agricultural and Environmental Applications.

[fsn31135-bib-0021] Sivieri, K. , Freire, F. C. , Lopes, N. P. , Shiraishi, C. T. D. , Pires, A. , Lima, A. C. D. , … Bianchi, F. (2017). Synbiotic yogurts and the elderly. Yogurt in Health and Disease Prevention, 259–271.

[fsn31135-bib-0022] Slozhenkina, M. , Druker, O. , Kryuchkova, V. , & Zlobina, E. (2018). Application possibilities of vegetable and prebiotic components in fermented milk products. 17th International Scientific Conference: Engineering for Rural Development: 497–507.

[fsn31135-bib-0023] Slozhenkina, M. , Serova, O. , Vodolazkova, M. , & Zlobina, E. (2017). Functional and technological characteristics of new cheese product with vegetable and prebiotic components. 16th International Scientific Conference: Engineering for Rural Development: 393‐401

[fsn31135-bib-0024] Sukhikh, S. A. , Krumlikov, V. Y. , Evsukova, A. O. , & Asyakina, L. K. (2017). Formation and study of symbiotic consortium of lactobacilli to receive a direct application starter. Foods and Raw Materials, 5, 51–62. 10.21179/2308-4057-2017-1-51-62

[fsn31135-bib-0025] Tamime, A. Y. , & Robinson, R. K. (2007). Tamime and Robinson's Yoghurt Science and technology (3rd, ed., XIII‐XVI). London, UK: Woodhead Publishing.

[fsn31135-bib-0026] Tari, A. I. N. , Handayani, C. B. , & Hartati, S. (2018). The characteristics of synbiotic yoghurt freeze‐drying supplemented by purple sweet potato (study on sucrose concentration as cryoprotectant). Proceedings of the International Conference on Applied Science and Engineering (ICASE 2018), 175, 45–47.

[fsn31135-bib-0027] The order of the Government of the Russian Federation dated 25.10.10 , # 1873‐p «Principles of State policy of the Russian Federation in the field of healthy nutrition of the population for the period till 2020» [Internet] Retrieved from: http://docs.cntd.ru/document/902242308. Data Access: April 24, 2019.

[fsn31135-bib-0028] Vasiljevic, T. , Kealy, T. , & Mishra, V. K. (2007). Effects of beta‐glucan addition to a Probiotic containing yogurt. Journal of Food Science, 72, C405–C411. 17995639 10.1111/j.1750-3841.2007.00454.x

[fsn31135-bib-0029] Vyas, U. , & Ranganathan, N. (2012). Probiotics, Prebiotics, and Synbiotics: Gut and Beyond. Gastroenterology Research and Practice. Article ID 872716 10.1155/2012/872716 PMC3459241 23049548

[fsn31135-bib-0030] Watson, R. R. , & Preedy, V. R. (2015). Probiotics, Prebiotics, and Synbiotics. Bioactive Foods in Health Promotion (1st ed.). Elsevier: Academic Press.

[fsn31135-bib-0031] Wattananapakasem, I. , van Valenberg, H. J. F. , Fogliano, V. , Costabile, A. , & Suwannaporn, P. (2018). Synbiotic microencapsulation from slow digestible colored rice and its effect on yoghurt quality. Food and Bioprocess Technology, 11, 1111–1124. 10.1007/s11947-018-2068-7

[fsn31135-bib-0032] Winarti, S. , & Saputro, E. A. (2015). Physicochemical and organoleptic properties of dried synbiotics yoghurt from lesser yam tubers (*Dioscoreaesculenta *L.). Bali, Indonesia: 3rd Bali International Seminar on Science & Technology (BISSTECH)

[fsn31135-bib-0033] Young, J. (1998). European market developments in prebiotic‐ and probiotic‐containing foodstuffs. British Journal of Nutrition, 80, S231–S233. 10.1017/S0007114500006085 9924290

